# Supplementary material for: Allosteric regulation and crystallographic fragment screening of SARS-CoV-2 NSP15 endoribonuclease
Source: Nucleic Acids Res. 2023 Apr 28;51(10):5255–70. doi: 10.1093/nar/gkad314 (PMC10250223; doi:10.1093/nar/gkad314)
Supplement: gkad314_Supplemental_Files [file gkad314_supplemental_files.zip › Supplementary datasets.zip!/160/Refine_8/01-BUSTER/Cycle-1/shell.01/R_Rfree.html]

# Rxpct factors in resolution bins at cycle 0


|  |  |  |  |  |  |  |  |  |
| --- | --- | --- | --- | --- | --- | --- | --- | --- |
|  | **<d> (Å)**  Rxpct\_Work | Rxpct\_Free | Rxpct\_All | |  |  |  |  |
|  | 14.6046 | 0.3722 | 0.5623 | 0.3788 |  |
|  | 8.4320 | 0.2388 | 0.2680 | 0.2405 |  |
|  | 6.5314 | 0.2214 | 0.2586 | 0.2235 |  |
|  | 5.5200 | 0.1978 | 0.2390 | 0.1994 |  |
|  | 4.8682 | 0.1637 | 0.2017 | 0.1658 |  |
|  | 4.4035 | 0.1674 | 0.1889 | 0.1686 |  |
|  | 4.0506 | 0.1719 | 0.1959 | 0.1730 |  |
|  | 3.7709 | 0.1801 | 0.2528 | 0.1840 |  |
|  | 3.5421 | 0.1815 | 0.2192 | 0.1834 |  |
|  | 3.3505 | 0.1879 | 0.2545 | 0.1915 |  |
|  | 3.1870 | 0.1920 | 0.2527 | 0.1954 |  |
|  | 3.0453 | 0.1962 | 0.2436 | 0.1983 |  |
|  | 2.9209 | 0.2048 | 0.2114 | 0.2051 |  |
|  | 2.8107 | 0.1960 | 0.2493 | 0.1985 |  |
|  | 2.7120 | 0.2010 | 0.2128 | 0.2016 |  |
|  | 2.6231 | 0.2073 | 0.2305 | 0.2087 |  |
|  | 2.5423 | 0.2130 | 0.2212 | 0.2135 |  |
|  | 2.4686 | 0.2173 | 0.2175 | 0.2173 |  |
|  | 2.4010 | 0.2242 | 0.2376 | 0.2248 |  |
|  | 2.3386 | 0.2515 | 0.2764 | 0.2526 |  |
|  | **Overall** | 0.2000 | 0.2390 | 0.2020 |  |

# Rxpct factors in resolution bins at cycle 1


|  |  |  |  |  |  |  |  |  |
| --- | --- | --- | --- | --- | --- | --- | --- | --- |
|  | **<d> (Å)**  Rxpct\_Work | Rxpct\_Free | Rxpct\_All | |  |  |  |  |
|  | 14.6046 | 0.3719 | 0.5602 | 0.3785 |  |
|  | 8.4320 | 0.2381 | 0.2664 | 0.2398 |  |
|  | 6.5314 | 0.2201 | 0.2572 | 0.2222 |  |
|  | 5.5200 | 0.1954 | 0.2360 | 0.1971 |  |
|  | 4.8682 | 0.1615 | 0.1983 | 0.1635 |  |
|  | 4.4035 | 0.1649 | 0.1919 | 0.1664 |  |
|  | 4.0506 | 0.1692 | 0.1957 | 0.1704 |  |
|  | 3.7709 | 0.1773 | 0.2522 | 0.1813 |  |
|  | 3.5421 | 0.1798 | 0.2125 | 0.1815 |  |
|  | 3.3505 | 0.1856 | 0.2504 | 0.1891 |  |
|  | 3.1870 | 0.1910 | 0.2508 | 0.1943 |  |
|  | 3.0453 | 0.1954 | 0.2386 | 0.1973 |  |
|  | 2.9209 | 0.2040 | 0.2098 | 0.2042 |  |
|  | 2.8107 | 0.1957 | 0.2478 | 0.1981 |  |
|  | 2.7120 | 0.2017 | 0.2219 | 0.2028 |  |
|  | 2.6231 | 0.2085 | 0.2316 | 0.2098 |  |
|  | 2.5423 | 0.2135 | 0.2240 | 0.2141 |  |
|  | 2.4686 | 0.2189 | 0.2165 | 0.2188 |  |
|  | 2.4010 | 0.2250 | 0.2368 | 0.2255 |  |
|  | 2.3386 | 0.2549 | 0.2822 | 0.2562 |  |
|  | **Overall** | 0.1986 | 0.2378 | 0.2006 |  |

# Rxpct factors in resolution bins at cycle 2


|  |  |  |  |  |  |  |  |  |
| --- | --- | --- | --- | --- | --- | --- | --- | --- |
|  | **<d> (Å)**  Rxpct\_Work | Rxpct\_Free | Rxpct\_All | |  |  |  |  |
|  | 14.6046 | 0.3798 | 0.5709 | 0.3865 |  |
|  | 8.4320 | 0.2436 | 0.2678 | 0.2450 |  |
|  | 6.5314 | 0.2213 | 0.2577 | 0.2233 |  |
|  | 5.5200 | 0.1951 | 0.2401 | 0.1969 |  |
|  | 4.8682 | 0.1591 | 0.2015 | 0.1615 |  |
|  | 4.4035 | 0.1626 | 0.1942 | 0.1643 |  |
|  | 4.0506 | 0.1671 | 0.1935 | 0.1683 |  |
|  | 3.7709 | 0.1757 | 0.2498 | 0.1796 |  |
|  | 3.5421 | 0.1796 | 0.2175 | 0.1815 |  |
|  | 3.3505 | 0.1848 | 0.2467 | 0.1882 |  |
|  | 3.1870 | 0.1904 | 0.2477 | 0.1936 |  |
|  | 3.0453 | 0.1952 | 0.2415 | 0.1972 |  |
|  | 2.9209 | 0.2041 | 0.2064 | 0.2042 |  |
|  | 2.8107 | 0.1963 | 0.2524 | 0.1989 |  |
|  | 2.7120 | 0.2008 | 0.2191 | 0.2017 |  |
|  | 2.6231 | 0.2069 | 0.2260 | 0.2081 |  |
|  | 2.5423 | 0.2122 | 0.2176 | 0.2125 |  |
|  | 2.4686 | 0.2169 | 0.2125 | 0.2167 |  |
|  | 2.4010 | 0.2235 | 0.2360 | 0.2241 |  |
|  | 2.3386 | 0.2523 | 0.2787 | 0.2535 |  |
|  | **Overall** | 0.1984 | 0.2380 | 0.2004 |  |

# Rxpct factors in resolution bins at cycle 3


|  |  |  |  |  |  |  |  |  |
| --- | --- | --- | --- | --- | --- | --- | --- | --- |
|  | **<d> (Å)**  Rxpct\_Work | Rxpct\_Free | Rxpct\_All | |  |  |  |  |
|  | 14.6046 | 0.3827 | 0.5795 | 0.3896 |  |
|  | 8.4320 | 0.2410 | 0.2731 | 0.2429 |  |
|  | 6.5314 | 0.2088 | 0.2601 | 0.2116 |  |
|  | 5.5200 | 0.1813 | 0.2333 | 0.1834 |  |
|  | 4.8682 | 0.1493 | 0.1960 | 0.1519 |  |
|  | 4.4035 | 0.1529 | 0.1847 | 0.1547 |  |
|  | 4.0506 | 0.1595 | 0.1863 | 0.1607 |  |
|  | 3.7709 | 0.1676 | 0.2517 | 0.1721 |  |
|  | 3.5421 | 0.1727 | 0.2117 | 0.1746 |  |
|  | 3.3505 | 0.1770 | 0.2477 | 0.1808 |  |
|  | 3.1870 | 0.1812 | 0.2397 | 0.1845 |  |
|  | 3.0453 | 0.1838 | 0.2270 | 0.1857 |  |
|  | 2.9209 | 0.1922 | 0.2053 | 0.1928 |  |
|  | 2.8107 | 0.1877 | 0.2388 | 0.1901 |  |
|  | 2.7120 | 0.1905 | 0.2214 | 0.1921 |  |
|  | 2.6231 | 0.1991 | 0.2150 | 0.2001 |  |
|  | 2.5423 | 0.2091 | 0.2239 | 0.2099 |  |
|  | 2.4686 | 0.2118 | 0.2233 | 0.2124 |  |
|  | 2.4010 | 0.2224 | 0.2294 | 0.2227 |  |
|  | 2.3386 | 0.2502 | 0.2598 | 0.2507 |  |
|  | **Overall** | 0.1903 | 0.2346 | 0.1925 |  |
